# Association of health literacy with cancer survival: a single-centre prospective cohort study

**DOI:** 10.2340/1651-226X.2025.42557

**Published:** 2025-04-02

**Authors:** Niclas Sandström, Antti Jekunen, Mikael Johansson, Heidi Andersén

**Affiliations:** a Cancer Clinic, Vaasa Central Hospital, The Wellbeing Services County of Ostrobothnia, Vaasa, Finland; b Faculty of Medicine, University of Turku, Turku, Finland; c Department of Diagnostics and Intervention Oncology, Umeå University Hospital, Umeå, Sweden; d Faculty of Medicine and Health Technology, Tampere University, Tampere, Finland

**Keywords:** socioeconomic status, patient-related factors, health literacy, health equity, health-related outcome

## Abstract

**Background and purpose:**

Health literacy is defined as the ability to find, understand and use health information for informed decision. The role of health literacy in treatment decisions and outcome remains largely unexplored. This study sought out to assess the effect of individual health literacy on overall survival (OS) in cancer patients in Ostrobothnia.

**Material and methods:**

The present study is a follow-up of a cross-sectional survey study performed during December 2021 and March 2022. The survey assessed socioeconomic factors, lifestyle factors and self-reported health literacy. The follow-up included data on recorded death, cause of death, performance status (PS), clinical frailty scale, Charlson comorbidity index and body mass index. The sample size for this study was 400 participants, and any participant with a malignancy was eligible for the study.

**Results:**

Low health literacy was associated with increased risk of death. The disparity remained after adjustments for age, sex, comorbidities, PS, stage and hazard ratios (HR) = 1.47 (1.01–2.14). After adjustments for lifestyle patterns, the disparity remained, HR = 1.49 (1.03–2.17). The difference diminished after adjustments for cancer types. The median OS was 3.6 months longer for those with medium-high health literacy than those with low health literacy.

**Interpretation:**

The results indicated health literacy having a direct, clinically significant, effect on OS, which is likely not explained by differences in cancer entity alone. Future studies should focus on assessing whether an intervention aiming to improve health literacy may improve overall cancer survival.

## Introduction

As the global burden of cancer increases, mainly due to increased life expectancy, the access to modern cancer care for different populations needs to be addressed. With an increased cancer prevalence, the strain on public health care systems increases, and the risk for different socioeconomic sub-populations increases. The access to modern cancer treatment is known to be negatively affected by low socioeconomic status (SES) [1]. This is known to result in worse treatment outcomes due to low SES, regardless of cancer type [2–6]. This disparity is well established and remains present as of today, also in high-income countries [7]. Health literacy has been proposed as a mediator of SES and health status, with high health literacy favouring better health outcomes [8]. Health literacy was defined by the World Health Organisation in the 1998 health promotion glossary as ‘The cognitive and social skills which determine the motivation and ability of individuals to gain access to understand and use information in ways which promote and maintain good health’ [9]. Low health literacy may be defined as the difficulty in accessing, understanding and using health information, which can result in challenges in making informed health decision and effectively managing personal health. High health literacy, on the other hand, enables individuals to make informed decisions and take an active role in managing their health. Our hypothesis is that high health literacy is associated with better cancer survival, due to individuals making informed decisions and taking an active role in managing their health.

Tools to investigate individual-level health literacy have been developed, for example, the European health literacy survey, which identified 47% of the study population having limited health literacy [10]. The comprehensive survey questionnaire was later challenged by Finbråten et al. [11], suggesting that a shorter survey questionnaire may be used. In 2020, health literacy and numeracy were assessed with one single sentence each, with a fifth reporting limited health literacy and numeracy [12]. Low health literacy, in turn, has been related to poorer outcome in the form of increased mortality [13]. The study population consisted of participants aged 50 and above, as part of a cohort studying health and ageing. Confirming results were reported by Berkman et al. [14] in a systematic review, suggesting poorer health outcomes and reduced use of healthcare services.

In cancer diseases, the role of health literacy has been investigated in the context of adherence to cancer screening programmes, shared decision-making and survivorship aspects [15–17]. The role of health literacy for cancer mortality remains largely unexplored, to our knowledge. In 2015, the relationship of health literacy and outcome was explored in colorectal cancers. The authors’ results indicated health literacy having no relationship with all-cause mortality [18]. On the other hand, Heudel et al. [19] conducted a retrospective study assessing how low e-health literacy affected overall survival (OS). The study indicated an association between low health literacy and OS, though confounders such as SES were not considered in the study. It remains unanswered what association might exist after adjusting for confounding factors. Health literacy may serve as a modifiable factor to improve health outcomes in cancer care.

This study aims to explore the role of patient-related factors and self-reported health literacy, on OS amongst any cancer type that participated in a survey during December 2021 and March 2022.

## Materials and methods

### Study design and participants

A community-based study was previously performed at the Cancer Clinic of Vaasa Central Hospital, Finland, during the period 20 December 2021–18 March 2022. The participants gave written informed consent and were distributed a questionnaire consisting of 21 questions, addressing patient-related factors. The questionnaire addressed socioeconomic factors, lifestyle and self-perceived health literacy, as reported by McCleary et al. [12]. The health literacy question used was ‘How difficult is it for you to understand medical forms and information?’ Participants were asked to report the result on a scale from 0 to 100, ranging from difficult to easy. The survey was distributed to any cancer patient attending the cancer clinic during the study period, this included patients admitted for radiotherapy treatment-planning, patients undergoing active oncological treatment, as well as patients in regular follow-up after treatment. After completion of the questionnaire, the treating physician added details about the cancer disease, including primary tumour origin, pathology report, Tumour (T), Nodes (N), Metastases (M)-staging (TNM) and WHO cancer stage. Patients with terminal stage disease and short life expectancy were not included in the study due to ethical considerations.

The present follow-up of the study was performed during May 2024. The follow-up information was collected from patient record files and was collected systematically in a database (MS Excel, Microsoft, Redmond, WA). The follow-up information included participation date, date of recorded death, survival time, cause of death, WHO performance status (PS), Clinical Frailty Scale (CFS), Charlson comorbidity index (CCI) and body-mass index (BMI). Deaths that occurred outside the well-being services county of Ostrobothnia were classed as unknown due to the cause of death not being verifiable.

### Statistical analyses

Analyses were conducted using the Statistical Package for the Social Sciences (SPSS) statistics version 28.0 (IBM Corp, Armonk, NY). Statistical significance was defined using two-tailed tests with a *p*-value < 0.05 considered significant. Confidence intervals of 95% were used. OS was defined as the time-period between inclusion in the study and death. Demographical characteristics were analysed using frequencies. Health literacy scores were divided in quartiles (Q), and Q1 (score <37) was defined as low health literacy. Medium health literacy was defined as Q2–Q3 (score 37–73) and high health literacy as Q4 (score >73). To compare low health literacy (Q1) and medium-high health literacy (Q2–Q4), crosstabulation analyses were used, and statistical significance analysed using Pearson correlation. When analysing survival estimate medians, Mantel-Cox analysis was used to assess statistical significance. Cox-regression analyses were used first for crude analyses to identify significant parameters that affect survival. The analyses were performed on socioeconomic variables, lifestyle variables and comorbidities. For sensitivity and specificity analyses, the ROC-curve was chosen, and C-statistic was defined as the area under the curve. Adjusted Cox-regressions were performed with different models using factors identified as significant in crude analyses. The models were adjusted to assess whether confounders affected the impact of health literacy on OS. For survival estimates of OS as a function of health literacy, the Kaplan-Meier estimate was used.

### Ethical considerations

This study was approved by the Vaasa Central Hospital (VKS_2021_42_JYL). All individual participants provided written-informed consent. The Southwest Finnish ethics committee approved this study because this study does not involve interventions, acknowledged by study subjects. General Data Protection Regulation (EU) 2016/679 was followed. This study was conducted according to the 1964 Helsinki declaration and its later amendments. The study design was a community-based research, and it was approved by both the community and the Cancer Association of Ostrobothnia. In a conducted workshop, the research questions were found to be in the interest of the community.

## Results

### Baseline characteristics

401 participants participated in this study, of which 52% were males, and one was lost to follow-up (Table 1). The median age for the study population was 70. Patients with WHO cancer stage IV were 49.1% of the study population. Regarding PS, a minority (3.3%) were defined as PS 3–4. When assessing fitness in the form of frailty, the majority of the study population (51.0%) were defined as 3–5 according to CFS. The median BMI for the study population was 25.5. Most participants (57.8%) had no comorbidities according to CCI. The median OS for the study population was 835.0 days. In total, 154 participants died during follow-up. Cancer-specific deaths were the most common cause of death (89.6%), and in 7.1% of cases, the cause of death was unknown. The median self-reported health literacy was 53, Q1 being <37.

**Table 1:** Baseline characteristics of the study population.

Variable	All cancer types	
	*N*	%
**Sex**		
Male	208	52
Female	192	48
**Age**		
Median (Q1, Q3)	70 (61, 76)	
**Stage**		
I–III	196	50.9
IV	189	49.1
**Performance status**		
0–2	387	96.8
3–4	13	3.3
**Clinical frailty scale**		
Robust (1–2)	148	37.0
Pre-frail (3–4)	204	51.0
Frail (≥ 5)	48	12.0
**BMI**		
Median (Q1, Q3)	25.45 (23, 29)	
**Charlson comorbidity index score**		
0	231	57.8
1–2	146	36.5
3–4	17	4.3
≥ 5	6	1.5
**Survival, days**		
Median (Q1, Q3)	835.0 (428.5, 863.0)	
**Cause of death**		
Cancer specific	138	89.6
Other cause	5	3.2
Unknown	11	7.1
**Self-reported health literacy**		
Median (Q1, Q3)	52.50 (37, 73)	

Q: quartile; BMI: Body-mass index.

### Comparison of low health literacy and medium-high health literacy

When comparing low health literacy and medium-high health literacy groups (Table 2), the majority in the low category were males (64.5%). In the medium-high health literacy population, females were slightly more common (51.9%). The median age in the low health literacy group was 73, as opposed to 68 in the medium-high health literacy group. The prevalence of stage IV was higher in the low health literacy group (58.6%) as compared to the medium-high health literacy group (45.5%, *p* = 0.032). There was a nonsignificant trend in PS, BMI and CCI, whereas in CFS, a significant difference was observed. Low health literacy had a lower proportion of robust participants (20.4%), as compared to medium-high health literacy (43.5%, *p* < 0.001). Regarding OS, low health literacy had a poorer median OS of 607 days, compared to medium-high health literacy with a median OS of 718 days.

**Table 2:** Comparison of self-reported health literacy groups Q1 (low) versus Q2–Q4 (medium to high).

Health literacy	Q1	Q2–Q4	*P*
	*N* (%)	*N* (%)	
**Sex**			*p* = 0.006
Male	60 (64.5%)	136 (48.1%)	
Female	33 (35.5%)	147 (51.9%)	
**Age**			*p* = 0.032
Median (Q1, Q3)	73 (66, 79)	68 (59, 75)	
**Cancer types**			*p* = 0.002
Breast	10 (11.9%)	74 (88.1%)	
Lung	21 (33.9%)	41 (66.1%)	
Gastrointestinal	25 (37.9%)	41 (62.1%)	
Prostate	12 (19.0%)	51 (81.0%)	
Other	25 (24.8%)	76 (75.2%)	
**Stage**			*p* = 0.032
I–III	36 (41.4%)	150 (54.5%)	
IV	51 (58.6%)	125 (45.5%)	
**PS**			*p* = 0.364
0–2	89 (95.7%)	276 (97.5%)	
3–4	4 (4.3%)	7 (2.5%)	
**Clinical frailty scale**			*p* < 0.001
Robust (1–2)	19 (20.4%)	123 (43.5%)	
Pre-frail (3–4)	54 (58.1%)	137 (48.4%)	
Frail (≥ 5)	20 (21.5%)	23 (8.1%)	
**BMI**			*p* = 0.464
Median (Q1, Q3)	24.9 (23.0, 27.6)	25.7 (23.0, 28.8)	
**Charlson comorbidity index score**			*p* = 0.164
0	47 (50.5%)	175 (61.8%)	
1–2	41 (44.1%)	95 (33.6%)	
3–4	3 (3.2%)	11 (3.9%)	
≥ 5	2 (2.2%)	2 (0.7%)	
**Survival, days**			*p* < 0.001
Estimate (lower limit, upper limit)	607 (542, 672)	718 (685, 752)	

Q: quartile; PS: Performance status; BMI: Body-mass index.

### Crude cox-regression analyses of self-reported health literacy and survival

Table 3 presents unadjusted hazard ratios (HRs) of socioeconomic variables affecting survival. Factors that had a statistically significant effect on survival were age, sex, cancer type and cancer stage. Mother tongue, education, occupation, affording a sudden payment of 1,200 €, income and relationship status did not affect survival significantly. Age had a HR of 1.02 (95% CI 1.01–1.04), and male sex 1.40 (95% CI 1.01–1.93). Regarding cancer characteristics, lung cancer (HR = 5.55) and advanced disease (HR = 4.78) were associated with increased hazard. Table 4 displays unadjusted analyses for lifestyle factors affecting survival. Factors that displayed statistical significance were smoking status; exposure to vapours, gas, dust and fumes (VGDF); dietary habits; exercise; and low health literacy. The HR for a patient with smoking habit increased with 1.52 (95% CI 1.10–2.10), and that of a patient exposure to VGDF increased by 1.46 (95% CI 1.06–2.00). 1–3 portions of greens in diet increased HR by 1.44 (95% CI 1.00–2.08). Not exercising according to recommendations increased HR by 2.43 (95% CI 1.69–3.48). Increased HR was observed in low health literacy (HR = 1.86 (95% CI 1.32–2.64)). Table 5 shows the impact of comorbidities on OS. PS 3–4 was associated with an increased HR of 7.24. High frailty (≥5) as classed by CFS was associated with increased HR = 11.31 (95% CI 6.81–18.78). CCI score 1–2 was associated with increased HR = 2.20 (95% CI 1.59–3.06). BMI was not associated with a significant difference in HR.

**Table 3:** Crude cox-regression analysis on the impact of socioeconomic variables on overall survival.

Variable	Crude analysis	
	HR	95% CI
**Language**		
Finnish	1.15	0.83–1.59
Swedish, ref	1	
**Age**	1.02	1.01–1.04
**Sex**		
Male	1.40	1.01–1.93
Female, ref	1	
**Cancer type**		
Lung	5.55	2.90–10.64
GI	4.81	2.49–9.26
Prostate	2.04	0.98–4.24
Other types	4.34	2.31–8.14
Breast, ref	1	
**Stage**		
IV	4.78	3.24–7.05
I–III, ref	1	
**Education**		
Primary	1.28	0.86–1.89
Secondary	0.96	0.65–1.42
Tertiary, ref	1	
**Occupation**		
1–2	1.26	0.90–1.77
3–4, ref	1	
**Afford payment**		
No	1.07	0.77–1.51
Yes, ref	1	
**Income/month**		
< 1,200 *€/month*	1.24	0.88–1.76
≥ 1,200 *€/month, ref*	1	
**Relationship status**		
Living alone	1.36	0.97–1.91
Living with someone, ref	1	

HR: Hazard ratio; 95% CI: 95% Confidence interval; GI: Gastrointestinal; Occupation classed using ISCO-08: International Standard Classification for Occupation.

**Table 4:** Crude cox-regression analysis on the impact of lifestyle factors on overall survival.

Variable	Crude analysis		C-statistics
	HR	95% CI	
**Smoking status**			
Ever-smoker	1.52	1.10–2.10	
Never-smoker, ref	1		
**Second-hand smoke**			
Yes	0.98	0.67–1.43	
No, ref	1		
**Exposure to VGDF**			
Yes	1.46	1.06–2.00	
No, ref	1		
**Exposure to asbestos**			
Yes	1.40	0.91–2.17	
No, ref	1		
**Alcohol**			
Consumes	0.63	0.46–0.87	
Does not consume, ref	1		
**Greens in diet**			
1–3 portions	1.44	1.00–2.08	
4–6 portions, ref	1		
**Activity per day**			
1–2 hours	1.25	0.89–1.76	
3–6 hours, ref	1		
**Exercise**			
No	2.43	1.69–3.48	
Yes, ref	1		
**Health literacy**			0.44
Low	1.86	1.32–2.64	
Medium-high, ref	1		

HR: Hazard ratio; 95% CI: 95% Confidence interval; VGDF: Vapours, gas, dust and fumes.

**Table 5:** Crude cox-regression analysis on the impact of comorbidities on overall survival.

Variable	Crude analysis	
	HR	95% CI
**Performance status**		
3–4	7.24	3.91–13.44
0–2, ref	1	
**Clinical frailty scale**		
Frail (≥ 5)	11.31	6.81–18.78
Pre-Frail (3–4)	3.20	2.06–4.99
Robust (1–2), ref	1	
**CCI-score**		
≥ 5	2.02	0.64–6.42
3–4	1.67	0.77–3.64
1–2	2.20	1.59–3.06
0, ref	1	
**BMI**	0.96	0.90–1.03

HR: Hazard ratio; 95% CI: 95% Confidence interval; CCI: Charlson comorbidity index; BMI: Body-mass index.

### Survival models

**Figure 1:**
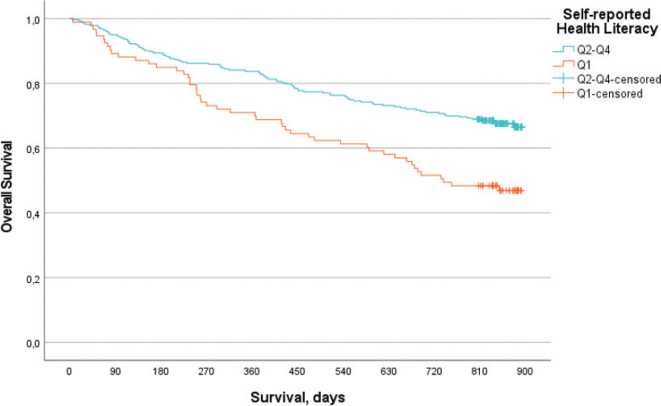
Kaplan-Meier estimate of overall survival comparing health literacy Q1 and Q2–Q4, p < 0.001.

Using the HR observed in crude analyses, adjusted hazard ratio (aHR) models were formed (Table 6). Models 1–3 display statistical significance, with increased aHR, when adjusted for age, sex, CCI, PS and cancer stage. Model 4 is adjusted for age, sex, CCI, PS, cancer stage and cancer types. The model indicates increased hazard (aHR = 1.33); however, no statistical significance is observed (95% CI 0.91–1.94). A sensitivity analysis was performed, excluding lung cancer participants. Model 5 is adjusted for age, sex, CCI, PS, cancer stage and cancer types (lung cancer excluded). An increased hazard (aHR = 1.57) with statistical significance was observed (95% CI 1.01–2.44). Model 6 presents the impact of low health literacy on OS when adjusted for lifestyle patterns. The model is adjusted for age, sex, smoking status, dietary habits and exercise and displays an increased hazard (aHR = 1.49) with statistical significance (95% CI 1.03–2.17). Figure 1 is a Kaplan-Meier estimate of OS as a function of health literacy. The figure shows that low health literacy was associated with shorter OS when compared to medium to high health literacy.

**Table 6:** Adjusted cox-regression models for the impact of Q1 health literacy on overall survival.

Regression model	aHR	Lower limit	Upper limit	Adjusted for
**Model 1**	1.67	1.17	2.38	Age, sex
**Model 2**	1.62	1.13	2.32	Age, sex, CCI, PS
**Model 3**	1.47	1.01	2.14	Age, sex, CCI, PS, stage
**Model 4**	1.33	0.91	1.94	Age, sex, CCI, PS, stage, cancer types
**Model 5**	1.57	1.01	2.44	Age, sex, CCI, PS, stage, cancer types (lung cancer excluded)
**Model 6**	1.49	1.03	2.17	Age, sex, smoking status, dietary habits, exercise

aHR: adjusted hazard ratio; CCI: Charlson Comorbidity Index; PS: Performance Status.

## Discussion

This study sought to explore the role of low health literacy on OS in cancer patients in Ostrobothnia, Finland. The results indicate low health literacy being an independent factor affecting survival, and the disparity remained after adjustments for age, sex, comorbidities, PS and cancer stage. The disparity could no longer be observed after adjustment for cancer type. After adjustments for lifestyle factors, an increased aHR remained. Participants with self-reported medium to high health literacy lived 3.6 months longer than those with low health literacy, with an aHR of Model 3, after adjusting for age, sex, stage, CCI and PS.

The results of our study indicate low health literacy being an independent factor affecting survival. The impact of health literacy on cancer survival was previously explored by Busch et al. [18] who reported that health literacy did not affect survival significantly. This study was limited due to a limited proportion of participants with marginal or inadequate levels of health literacy. An important factor affecting OS is the rate of guideline adherence. Several studies have demonstrated improved survival when treatment is given according to guidelines. In breast cancer, improved survival was observed when treatment was given in accordance with guidelines, and ethnicity had no effect on treatment choice [20, 21]. In gastric cancer and rectal cancer, guideline adherence was associated with improved OS; however, the rate of guideline adherence decreased with increasing age [22, 23]. In the population of Ostrobothnia, a previous study indicated patient-related factors, such as health literacy, having no effect on the rate of guideline adherence or treatment toxicity [24]. Furthermore, in Ostrobothnia, lung cancer treatment guideline adherence in the elderly was associated with increased OS [25]. Due to treatment being largely given according to guidelines in Ostrobothnia, we believe these results indicate decreased OS not being explained by non-adherence to treatment guidelines. We hypothesise that the difference could be explained by insufficient health literacy affecting shared decision-making. Due to insufficient health literacy, participants may be in poorer condition than self-reported. Ten years ago, this was explored by Serper et al. [26], who found that cognitive function in the elderly explained largely the relationship between low health literacy and poorer health status. If not acknowledged by healthcare staff members, treatment intensity may be incorrect, leading to poorer outcome.

The clinical usefulness of assessing health literacy was discussed in 2021 by an Australian study [27]. This study found no correlation between self-perceived health literacy and objectively assessed health literacy, largely due to inconsistent definitions of health literacy. To increase the accuracy, health literacy models should consider social, environmental and situational factors. Dahlgren and Whitehead [28] discussed the interplay between lifestyle factors, living and working conditions, and socioeconomic and environmental conditions in their rainbow model. Cultural and socioeconomic aspects affect both living and working conditions, which, in turn, affects lifestyle patterns. Low educational level is associated with low health literacy, and low health literacy is proposed to mediate the relationship between SES and both health status and lifestyle patterns [29, 30]. Our models consider statistically significant health status (in the form of PS, CFS, CCI and BMI) and lifestyle pattern (smoking status, dietary habits and exercise status) differences when assessing the impact of health literacy on OS. We believe our results suggest health literacy is an important parameter to consider in improving outcome of cancer management. In the present study, there was an improved median OS of more than 3 months, which is a clinically significant difference. In Finland, clinical significance is defined as an OS benefit of over 3 months. What possible causal connection might exist is beyond the scope of the present study.

In Finland, socioeconomic differences in cancers are documented. In *Cancer in Finland 2022*, by Pitkäniemi et al. [31], a national report on cancer, Chapter 14 discusses socioeconomic disparities in cancer incidence and mortality. In breast cancer and prostate cancer, higher education was associated with increased cancer incidence but with lower mortality. In lung cancer, lower education was associated with increased cancer incidence and higher cancer mortality. The disparity in lung cancer was the most notable. In Ostrobothnia, health literacy differences have been documented, of which lung cancer had the lowest reported health literacy [32]. Low health literacy is associated with poorer lifestyle patterns, and these factors may affect survival directly.

When adjusted for cancer types, the effect of low health literacy on OS diminished. Lung cancer was the cancer type with the greatest effect on survival, having an HR of 5.55. Socioeconomic differences in lung cancer have been reported in Nordic countries, regardless of histological subtype, and the disparity remains as of today [33]. The majority of lung cancer patients are diagnosed at late stage, and screening of high-risk populations (smokers) has demonstrated reduced mortality [34]. In Finland, however, lung cancer screening amongst high-risk populations is not yet implemented. Participation in screening is suggested to be hindered by low health literacy, and previous studies have indicated lung cancer patients having inadequate levels of health literacy [35, 36]. Our findings may, therefore, be of importance in the implementation of future lung cancer screening programs in order to increase screening participation in low health literacy populations. The effect of low health literacy on OS might have decreased after adjustments for cancer types, due to the prevalence of low health literacy in lung cancer patients and the greatest HR observed.

### Strengths and limitations

The strengths of this study include detailed patient-reported variables on SES, lifestyle patterns and health literacy, which may be lacking in patient records. This study included longitudinal follow-up combined with details of primary tumour origin, stage, comorbidities, PS and frailty. To reduce the confounding effect of cancer stage, PS and comorbidities, multivariate analyses were performed.

This study is primarily limited by the single-centre design and limited sample size. Furthermore, the design of the health literacy question was a one-item scale, and these are very error prone. The results of the present study should not be used as a predictive model. The C-statistics of our models suggest poor sensitivity and specificity for predicting OS, and the sample size was limited when performing sensitivity analyses for the different subgroups of cancer diseases. The model is useful in acknowledging a potential direct effect of low health literacy on OS. CFS was estimated retrospectively based on medical records and could, thus, be varying depending on the charting of medical personnel. This study did not record given treatment, and, thus the influence of treatment on OS may not be assessed by the present study.

## Conclusions

Limited health literacy may result in poorer cancer survival due to a gap in communication between patients and medical staff. Patients may be in poorer condition than self-reported, leading to incorrect treatment intensity with a worse outcome amongst patients with limited health literacy. Recognising this communication gap by medical staff is important to provide both correct and equitable healthcare. Future studies should consider whether interventions aimed at increasing health literacy may alleviate disparities in OS.

## Supplementary Material

Association of health literacy with cancer survival: a single-centre prospective cohort study

## Data Availability

Due to its proprietary nature data and Finnish General Data Protection Regulation, data cannot be made public; request should be addressed to Heidi Andersén.
