# Supplementary material for: Association of health literacy with cancer survival: a single-centre prospective cohort study
Source: Acta Oncol. 2025 Apr 2;64:42557. doi: 10.2340/1651-226X.2025.42557 (PMC11981010; doi:10.2340/1651-226X.2025.42557)
Supplement: Association of health literacy with cancer survival: a single-centre prospective cohort study [file AO-64-42557-s1.pdf]

Supplementary material has been published as submitted. It has not been copyedited, or typeset by Acta Oncologica

Supplementary material

1. What is your highest attained educational level?
2. What is your occupation?
3. Could you afford a sudden payment of €1200?
4. Is your income over €1200?
5. Are you living alone?
6. Have you smoked at any time?
7. If you have smoked, how many pack-years have you smoked?
8. Have you been exposed to second-hand smoke at home or at work?
9. Have you been exposed to vapours, gas, dust, and fumes at work?
10. Have you been exposed to asbestos?
11. Do you consume alcohol?
12. How many portions of greens in diet do you consume per day?
13. How active are you during the day?
14. Do you exercise according to national recommendations?

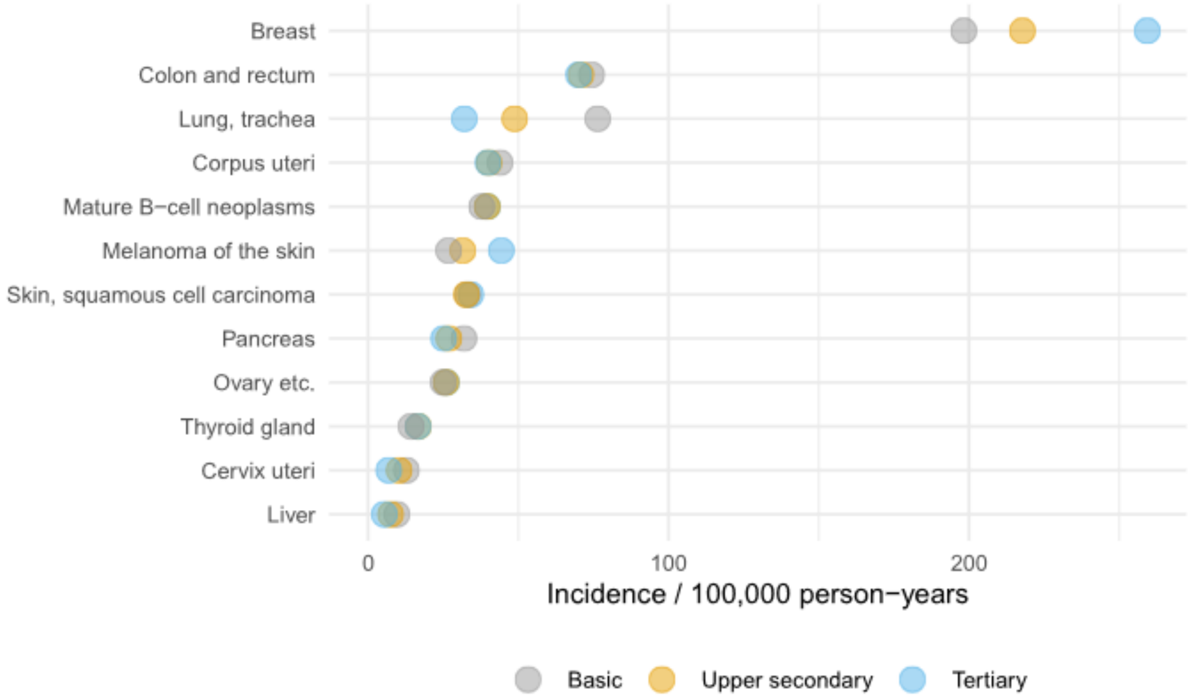

Figure 36: Incidence of cancer in women (per 100,000 person-years and age-standardised to the 2014 Finnish population) in the population aged over 25 by level of education in 2018–2022.

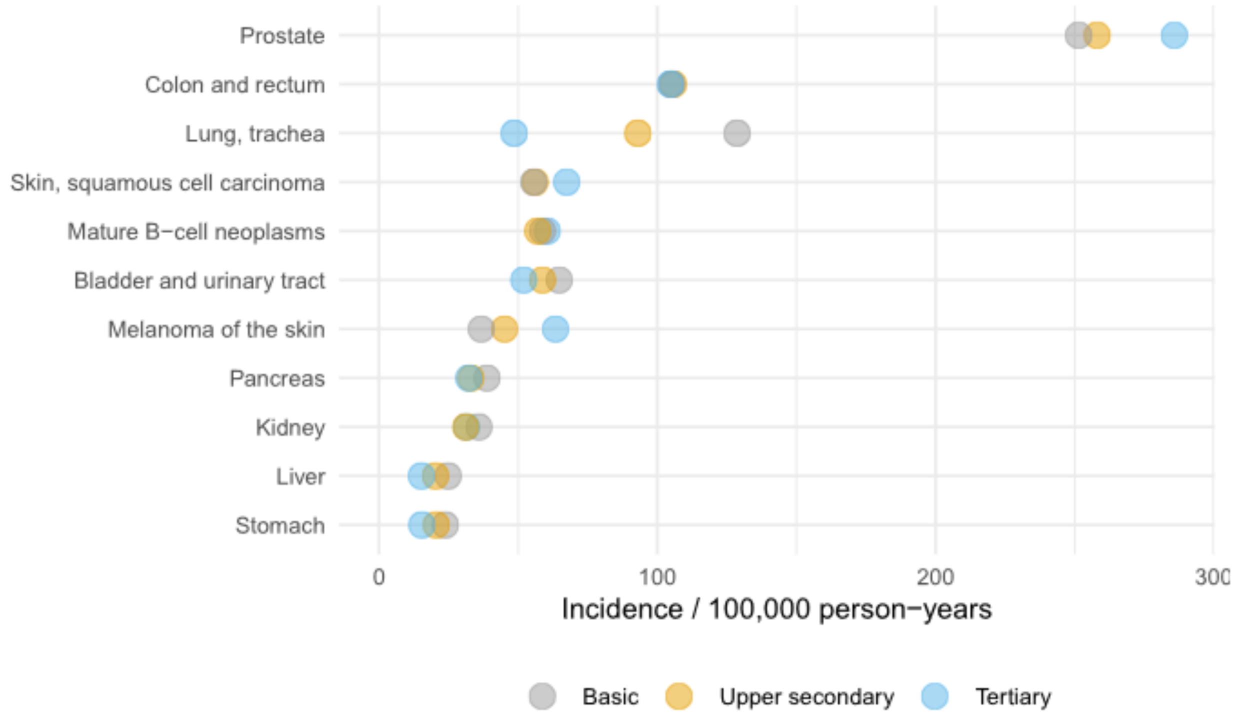

Figure 37: Incidence of cancer in men (per 100,000 person-years and age-standardised to the 2014 Finnish population) in the population aged over 25 by level of education in 2018–2022.

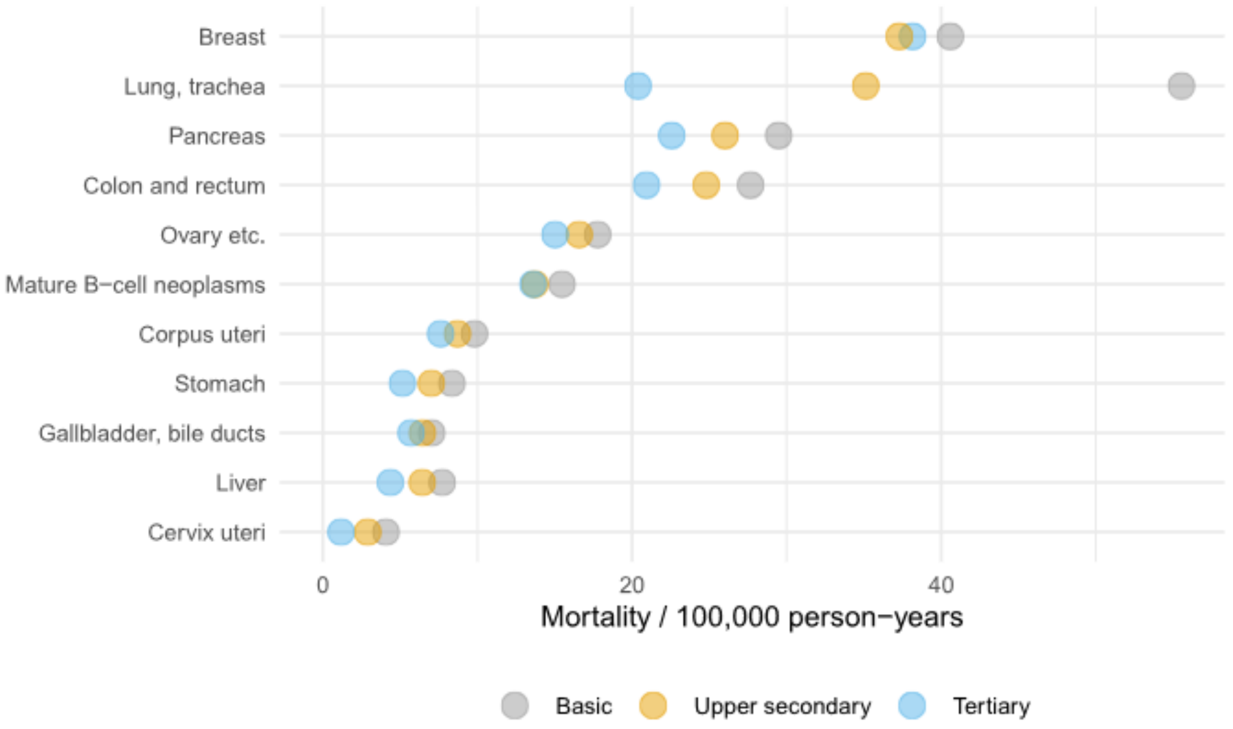

Figure 38: Cancer mortality in women (per 100,000 person-years and age-standardised to the 2014 Finnish pop- ulation) in the population aged over 25 by level of education in 2018–2022.

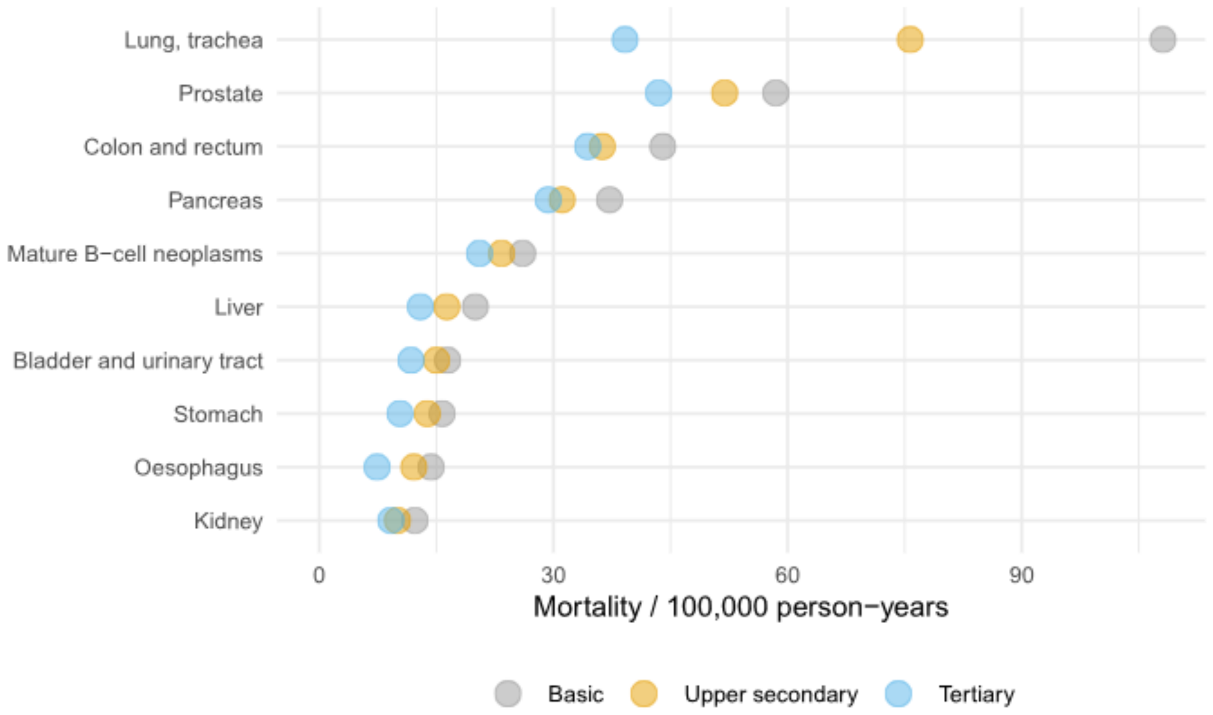

Figure 39: Cancer mortality in men (per 100,000 person-years and age-standardised to the 2014 Finnish popula- tion) in the population aged over 25 by level of education in 2018–2022.
